# Neuronal lineages derived from the nerve-associated Schwann cell precursors

**DOI:** 10.1007/s00018-020-03609-5

**Published:** 2020-08-03

**Authors:** Polina Kameneva, Maria Eleni Kastriti, Igor Adameyko

**Affiliations:** 1 Department of Physiology and Pharmacology, Karolinska Institute, Stockholm, 171 77 Sweden; 2 Department of Molecular Neurosciences, Center for Brain Research, Medical University Vienna, Vienna, 1090 Austria

**Keywords:** Schwann cell precursors, Peripheral nervous system, Chromaffin cells, Enteric nervous system, Neuroblastoma, Hirschsprung disease

## Abstract

For a long time, neurogenic placodes and migratory neural crest cells were considered the immediate sources building neurons of peripheral nervous system. Recently, a number of discoveries revealed the existence of another progenitor type—a nerve-associated multipotent Schwann cell precursors (SCPs) building enteric and parasympathetic neurons as well as neuroendocrine chromaffin cells. SCPs are neural crest-derived and are similar to the crest cells by their markers and differentiation potential. Such similarities, but also considerable differences, raise many questions pertaining to the medical side, fundamental developmental biology and evolution. Here, we discuss the genesis of Schwann cell precursors, their role in building peripheral neural structures and ponder on their role in the origin in congenial diseases associated with peripheral nervous systems.

## Introduction

Embryonic Schwann cell precursors (SCPs) are known as a progenitor population for myelinating and non-myelinating mature Schwann cells (SCs) covering the adult nerves. Indeed, generation of mature SCs can be seen as a well-known and canonical function of SCPs, which originate from neural crest cells (NCCs), a highly migratory and multipotent cell population that gives rise to many cell types in the body [1, 2]. During NCCs migration, a portion of the cells settles on the outgrowing sensory and motor peripheral nerves to become nerve-associated SCPs. SCPs express ERBB2/3 receptors, which recognize the ligand NRG1 on the surface of axons. This signaling pathway is necessary for the migration of SCPs along nerves as well as for the survival of SCPs and, indirectly, motor and sensory neurons (reviewed in [3]). In the case of *Erbb2/3* or *Nrg1* deficient mice, the ventral migration of the NCCs is disturbed, and, consequently, the sympathetic chain ganglia are hypoplastic—a finding reproduced in zebrafish [4, 5]. In these mice, SCPs are depleted from the motor and sensory nerves, which in turn degenerate in the absence of SCPs during late stages of embryonic development [6]. ERBB2/3-NRG1 signaling pathway additionally plays a role in differentiation of SCPs into myelinating and non-myelinating SCs. Large-diameter axons, which provide higher levels of NRG1 to SCPs, direct their differentiation towards myelinating SC, whereas the small-diameter axons become ensheathed into Remak bundles by non-myelinating SC [7, 8].

The story of the SCP origin starts with NCC migration, which is happening between E8.5 and E10.5 in the mouse, with SOX10^+^ NCCs taking different routes to their final and diverse destinations within the developing embryo. Around E9-9.5 and onwards, NCCs migrating next to the neural tube towards the dorsal aorta start to build the sympathetic chain and, with a slight delay, sensory neurons of the dorsal root ganglia. At the same time, the first motor axons start extending from the ventral portion of the neural tube. SCPs settle on these emerging autonomic, sensory and motor nerves to be guided to different tissues and organs of the developing vertebrate embryo. Recently, numerous studies recognized an unexpectedly broad developmental potential of SCPs in addition to their canonical role in generation of adult SC. The realization of such differentiation potential turned out to be dependent on a particular region, providing local tissues with necessary cell types [9]. Indeed, one of the early studies revealed that melanocytes (pigmented cells) largely originate from SCPs associated with the sensory innervation of the skin [10]. Lineage tracing experiments in mouse and chick embryos validated SCP-dependent origin of a large proportion of skin and hair follicle melanocytes. In zebrafish, post-neural crest progenitors residing in dorsal root ganglia and associated with peripheral nerves are also capable of producing melanocytes especially during metamorphosis [11]. These fish-specific FOXD3^+^/SOX10^+^ peripheral ganglia-associated cells might be considered homologous to the amniote SCPs [12].

Over the last decade, the emerging research on SCP multipotency clarified that SCPs give rise to a broad spectrum of cell types including parasympathetic neurons [13, 14], neurons of the enteric nervous system [15–17], chromaffin cells of adrenal medulla and extra-adrenal chromaffin organ of Zuckerkandl [18, 19], a portion of sympathetic neurons in lower trunk paraganglia [19] and glomus cells of the carotid body oxygen-sensing organ [20]. When it comes to derivatives of non-neural nature, SCP-dependent origin was confirmed for endoneural fibroblasts [21], mesenchymal stem cells of bone marrow [22], dental mesenchymal stem cells [23] and some chondrocytes [24]. These discoveries led to reconsidering SCPs as a multipotent population capable of producing seemingly unrelated cell types.

Taking in account all these findings, it might be hypothesized that peripheral neurogenesis has been partitioned by the evolution to either NCCs or SCPs operating as major neurogenic sources at the body periphery. The question that arises is why some neuronal populations are not generated directly by the migratory crest as in the case of sensory and sympathetic neurons. For instance, one of the reasons can be the location of the gangliogenesis in relation to the neural tube as well as the timing of neurogenesis. Dorsal root ganglia and sympathetic chain originate directly from NCCs at early stages and are located in a close proximity to the neural tube, because the embryo is still compact at this stage. At the same time, parasympathetic ganglia or the parts of enteric nervous system develop at a larger distance from dorsal neural tube, when the embryo undergoes intense growth at later, post-NCC stages. Besides, parasympathetic and enteric neurons chronologically appear in correlation with the development and growth of the organs they are going to innervate. The timing of the emergence of such structures can vary and include late stages of development—way beyond the NCC migration stage. As the embryo might be too large for the efficient migration and navigation of the crest cells, there might be a need for the long-lasting local neural crest cell-like source. This role is perfectly performed by the nerve-associated SCPs. The neuronal projections extending towards developing internal organs ensure the precision and efficiency of the SCP delivery [25].

Finally, instead of discussing why NCCs do not give rise to some neuronal populations via active migration, we might suggest the evolutionary concept, where neural crest dispersal is achieved via nerve-assisted delivery. Quite surprisingly, we can propose that the active migration of the NCCs can be, in fact, a later evolutionary acquisition relying on elaboration of complex and diverse navigation programs. At the same time, the basic ancient way of delivering progenitors might be rather represented by the SCP-like nerve-associated transportation route. Such nerve-assisted delivery of progenitors does not require the individualization of the navigation programs, which are necessary for the targeted active migration of progenitors to the sites, where specific neural and mesenchymal derivatives should form. The nerves, which possess ancient navigation tactics, can mediate all pathfinding and subsequently target all necessary regions of the body bringing multipotent cells on their surface. In places where needed, SCPs can detach and convert into other fates, whereas the rest will differentiate into mature SCs. Some support for such ideas comes from the recent discovery of the nerve-assisted delivery of SCPs to the lamprey gut, where these SCPs give rise to the enteric nervous system [26]. This suggests the ancient and widespread conserved role of SCPs being intermediates between the crest and the definitive cell type. Thus, SCPs might represent the “evolutionary early version” of the migratory neural crest and serve as a universal tool for the delivery of multipotent progenitors from the central nervous system to the body periphery.

## The developmental origin and basis of fate potential of SCPs

Even though it is clear that SCPs are neural crest-derived, the molecular mechanisms driving a portion of freely migrating NCCs into SCP-fate are not fully elucidated. This missing knowledge is fundamental to understand the mechanism driving the fate segregation towards nerve-associated SCPs prior to their differentiation towards different peripheral neuronal populations.

In an effort to address this key question, researchers used single-cell RNA sequencing (scRNA-seq) coupled to computational analysis of RNA velocity [25] on several stages of early murine neural crest development. The resulting model revealed that cell fate decisions in NCCs occur in a binary restrictive mode, where cells make a choice between two competing fate-specific programs at a time [27]. According to this model, fate decisions in the neural crest proceed via three distinct stages: initial co-activation of competing transcriptional programs, gradual biasing towards one of the programs under the pressure of intrinsic and extrinsic factors, followed by a commitment phase, when one of the programs dominates.

Soldatov and co-authors detected the presence of fate-biasing factors already during delamination, which suggests that the choice of a final fate might be influenced already at the level of the neural tube [27]. As NCCs delaminate and progress towards a migratory phase, one of the bifurcations provides the alternatives between sensory neuronal and common progenitors of autonomic and mesenchymal branches including SCP fate. Previous studies had revealed the role of DELTA-NOTCH signaling in the process of pro-neurogenic and non-neurogenic fate choice. Neuronal progenitors express DELTA-like1 signal, which acts on a NOTCH^+^ adjacent cells and inhibits their neuronal program, which leads them to adopting the glial program. This process, known as lateral inhibition, results in a salt-and-pepper induction pattern of the neuronal and glial cell types in dorsal root ganglia [28, 29]. The ablation of *Notch* from migrating NCCs causes severe hypoplasia of dorsal root ganglia and disturbed neuro-glial balance [30].

According to Soldatov et al., the second major fate split between neuroblasts and SCPs occurs within the autonomic nervous system domain. The authors of this study addressed the position and transcriptional signature of the neural crest subpopulation committing into SCPs, with *Zfp488, Mstn, Gpr17, Mpz* and other genes associated with this process. The position of forming SCPs among diversifying crest populations on the developmental trajectory has been verified by superimposing the more stagewise-advanced dataset containing unambiguous SCP cells. The commitment to the SCP fate might be triggered by the physical interaction with the nerves, DELTA-NOTCH signaling and higher levels of anchored NRG1 provided by the neurites quickly emerging from neuronal somas at this developmental stage. According to scRNA-seq data, all migratory NCCs express the NRG1 receptor *Erbb2/3*, which means that they must rely on some soluble NRG1 produced by a local source. This hypothesis is strengthened by the fact that in *Erbb3* knockout mice, there is a general neural crest deficiency both in the trunk and cranial parts [31].

Surprisingly, satellite glial cells forming inside of the ganglia are not sensitive to NRG1 signaling: in *Erbb2/3* knockouts they stay largely unaffected [10]. This suggests important differences in commitment tactics in ganglia-dwelling populations of peripheral glial cells. Even though it is increasingly clear that fate commitment in the SCP domain might be driven through extracellular signaling such as DELTA/NOTCH and NRG1/ERBB2/3, uncovering distinctions between developing satellite glial cells and SCPs along the outgrowing nerves is technically problematic. Despite the fact that Smart-seq2 single-cell transcriptomic data provide a good resource for studying molecular mechanisms of NCC commitment to the SCP fate, the cell numbers generated so far are not yet sufficient for the reliable analysis of fine transcriptional changes during SCP formation timeline. Such analysis is further complicated due to the extraordinary similarity of the molecular signature characterizing SCPs and migratory NCCs [27].

The maintenance of such similarity between NCCs and SCPs might hint us on the question about the molecular reasons of SCP multipotency. According to the existing data, SCPs and NCCs share major components of their gene regulatory networks, such as the transcriptional factors SOX10 and FOXD3. Research has uncovered similar gene expression regulation tactics between NCCs and SCPs, in particular in relation to regulation of *Sox10* expression, which is essential for NCC and SCP survival and differentiation [32]. Recent studies showed that maintenance of *Sox10* expression in NCCs and their differentiation towards SCPs as well as survival of SCPs and normal myelination additionally involve regulation by histone deacetylases HDAC1/2 [33–36]. HDAC1/2 interact with the *Sox10* enhancer regions *U1/MCS7* and *U3/MCS4* during the development of NCCs, SCPs and their glial fates. To maintain *Sox10* expression, HDAC2 activates *MCS4* and *Fabp7* indirectly and the *P0* promoter in a direct manner, further enhancing the expression of the glial program [33, 37]. NCCs and SCPs share the transcription factors PAX3, AP2A, and SOX10, which bind to the *U3/MCS4* enhancer. Therefore, SOX10 is involved in the control of its own expression, as well as being finely regulated by other transcriptional factors during the specification of SCPs [38]. The other common transcriptional factor found in both NCCs and SCPs, FOXD3, is indispensable for the maintenance of the migratory phenotype of NCCs and SCPs. Downregulation of FOXD3 plays an important role in the specification of melanocytes from both types of precursor cells [10, 39]. Therefore, the intimate relationships between SCPs and neuronal processes as well as survival of SCPs is established under the transcriptional control of a gene regulatory network shared with NCCs to a large extent.

Overall, the NCC-like multipotency of SCPs might reside in a common transcriptional code. However, the question of why SCPs give rise to particular derivatives in specific locations stays largely unexplored. We expect that this question will be addressed in the near future with the expansion of spatial transcriptomic techniques.

## SCP-dependent origin of parasympathetic and some sympathetic neurons

All daily functions in mammals result from the function of the two components of the peripheral nervous system: the parasympathetic and sympathetic divisions. The parasympathetic division of the peripheral nervous system is responsible for the so called “rest and digest” functions including salivation, digestion, urination and others. In contrast to this, the sympathetic system is responsible for the “fight or flight” response in periods of stress which includes increased heart rate, release of glucose into the bloodstream by the liver, dilated bronchioles facilitating increased breathing and alike. The important differences between parasympathetic and sympathetic neurons include neurotransmitter systems and relations to the location of the preganglionic fibers arriving from the central nervous system. Specifically, cholinergic parasympathetic ganglia are typically found within the target organ and receive preganglionic innervation via long axonal projections from the spinal cord. Contrary to this, sympathetic neurons are found further away from their targets to which they project longer axons as compared to parasympathetic neurons. Neurons of the sympathetic ganglia predominantly use norepinephrine as a neurotransmitter.

However, it is important to mention that it has been made increasingly clear that neurotransmitter expression alone is not enough to discriminate between parasympathetic and sympathetic ganglia. There are examples of cholinergic sympathetic innervation of sweat glands and periosteum also in line with the recent identification of cholinergic neuronal subtypes within stellate and thoracic sympathetic ganglia as revealed in a single-cell transcriptomic study [40–42].

Parasympathetic neurons are grouped into ganglia located in all parts of the body except for the limbs and other extremities. Particularly in the cranial and thoracic region of the body, parasympathetic neurons form the cranial ciliary, pterygopalatine, lingual, otic, submandibular ganglia and cardiac parasympathetic ganglia, all of which receive preganglionic innervation from preganglionic neurons of the brainstem through projecting cranial nerves [43, 44].

Early transplantation experiments and DiI tracing in chick embryos focusing on cranial regions had concluded that parasympathetic neurons originate from the migratory NCCs [45, 46]. However, soon it appeared obvious that active migration of cranial and trunk NCCs ceases to exist at E10 stage in mouse as by this time and all SOX10^+^ cells found to be nerve-associated [31]. Induction of genetic recombination in *Sox10*-*CreER*^*T2*^*/Rosa26*^*YFP*^ embryos at E11.5, a developmental point at which all SOX10^+^ cells are SCPs found on the nerves, labelled nearly all neurons in parasympathetic cranial ganglia and substantial part of caudal sympathetic ganglia analysed at E17.5 [13, 19]. Additionally, ASCL1^+^/PHOX2B^+^ cells, which represent committed parasympathetic progenitors, were revealed in close contact with preganglionic nerve fibers already at E12.5. At the same time, these nerve-associated pro-neural cells co-expressed SCP markers *Sox10* and *Erbb2/3* indicating that these progenitors are in transition from SCPs fate to parasympathetic fate at this developmental stage.

**Fig. 1:**
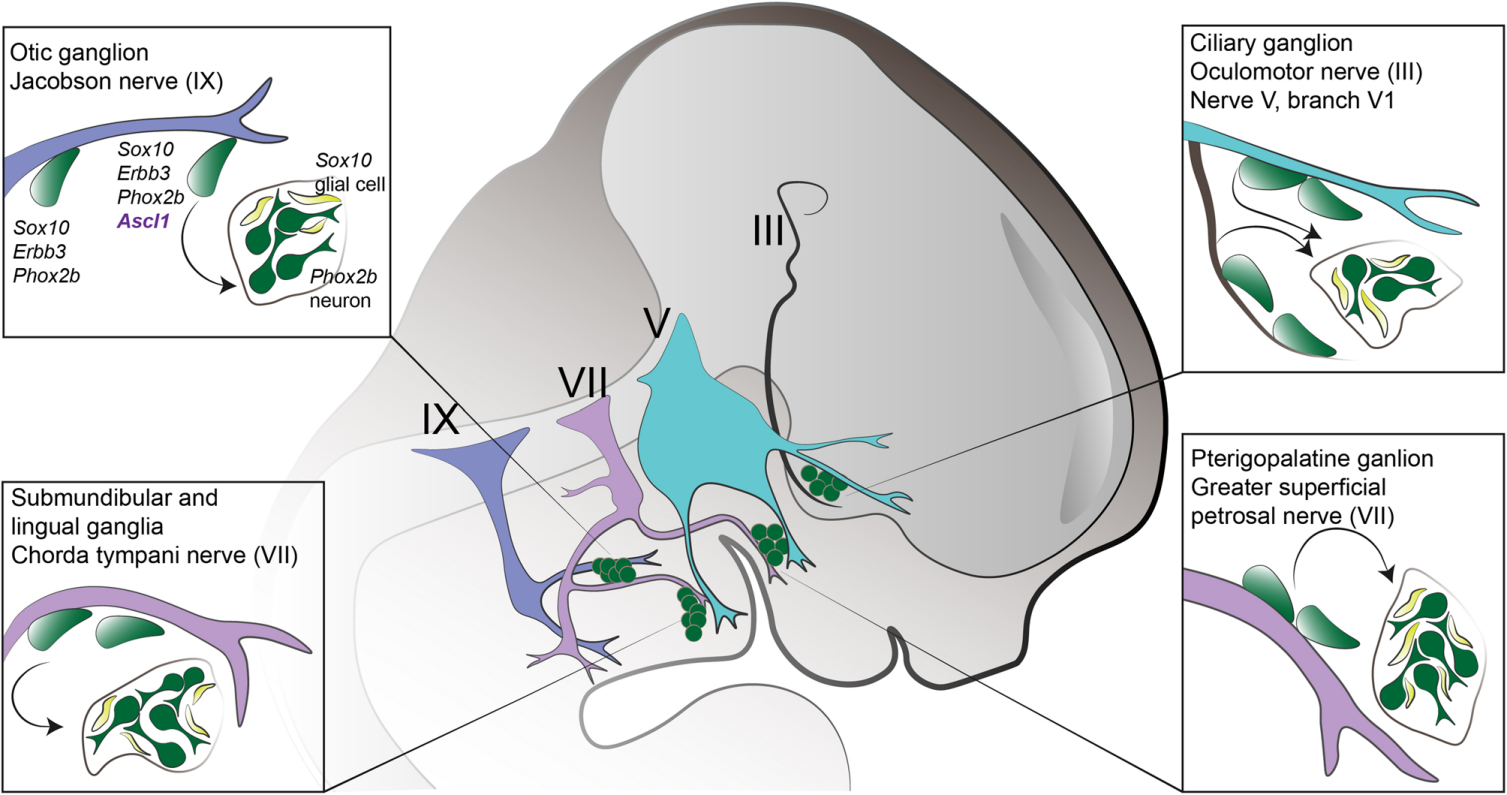
Neurons in parasympathetic ganglia in the cranial region: otic, submandibular, lingual, ciliary, and pterygopalatine originate from SCPs. SCPs first upregulate PHOX2B upon reaching the distal parts of the nerves, and upregulate ASCL1 and once the ganglia are formed the expression of SOX10 and PHOX2B becomes mutually exclusively restricted to glial and neuronal cell types respectively

Additionally, according to Espinosa-Medina and co-authors, SOX10^+^ SCPs found along the cranial nerves initially activate the expression of *Phox2b* during their migration towards the future locations of the parasympathetic ganglia. Then, upon their arrival to the location, where the ganglia will form, they activate the expression of the pro-neurogenic gene *Ascl1*. Shortly after their dissociation from the nerves and an onset of ganglia formation, the expression of *Sox10* and *Phox2b* becomes mutually exclusive. SOX10^+^ cells that remain associated with the axons lose expression of pro-neurogenic markers and commit to the Schwann cell lineage (Fig. 1) [14].

Apart from the lineage tracing experiments, a series of loss-of-function studies validated the SCP-based origin of parasympathetic neurons. For instance, *Ret*^−*/*−^ mice miss specific preganglionic visceral motor nerves (greater superficial petrosal and Jacobson’s nerves typically targeting developing parasympathetic ganglia in the head). The loss of these preganglionic nerves was associated with the absence of the corresponding parasympathetic ganglia they normally innervate, whereas the ganglia associated with the *chorda tympani* and oculomotor nerves developed normally, since these nerves are not affected by the loss of *Ret* [13]. Consistently, ablation of cranial nerves in *Neurog2*^−*/*−^ and *Neurog1*^−*/*−^; *Neurog2*^−*/*−^ mice showed a conceptually similar result, since the mutant embryos developed deficiencies of the major preganglionic parasympathetic nerves in the face and heart [14]. Furthermore, *Erbb3*^−*/*−^ mice are characterized by the absence of SOX10^+^ nerve-associated SCPs and are characterized by the absence of parasympathetic neurons. Finally, the analysis of *Ascl1*^−/−^ mice combined with tracing of the *Ascl1* lineage in *Ascl1*-*CreER*^*T2*^ embryos concluded that SCPs were not able to differentiate towards neurons due to the lack of this proneural gene, and, instead, *Ascl1*-deficient SCPs populated the preganglionic nerves while maintaining the expression of glial markers (SOX10, ERBB3, SOX2 and BFABP) [13]. Clonal lineage tracing analysis of SCPs contributing to parasympathetic neurons using a Confetti reporter revealed that a single SCP cell gives rise only to 2.5 parasympathetic neurons and 6-8 local glial cells on average, which suggests that large numbers of SCPs must be recruited to build parasympathetic ganglia, often hosting hundreds of neurons [13]. This observation renders SCPs as progenitors with limited expansion potential unlike classical stem cells with transiently amplifying cell cascade. Thus, local preganglionic nerves serve as important breeding centers ensuring sufficient numbers of SCPs, which allows the local preganglionic innervation to control the size of future parasympathetic ganglia.

Contrary to the case of cranial and cardiac parasympathetic ganglia, lineage tracing experiments in mice have shown that the vast majority of sympathetic neurons originates directly from the migratory NCCs. However, a small proportion of sympathetic neurons at the posterior part of the sympathetic chain appear to have a SCP origin [18]. Therefore, the possibility that SCPs and satellite glial cells indeed give rise to a fraction of sympathetic neuroblasts persists. In support of this fact, sympathetic paraganglia located in the vicinity of the developing adrenal gland and the organ of Zuckerkandl contain much higher proportion of SCP-derived sympathetic neurons—up to 13% and more if normalized for the recombination efficiency [19].

Until recently, lower trunk pelvic ganglia innervating the rectum, bladder and genitals were considered parasympathetic. This conclusion was supported by the early assumptions about sacral preganglionic neurons resembling preganglionic brainstem neurons in terms of their localization and pharmacology (being mainly cholinergic) and the fact that the function of said organs (rectum, bladder, genitals) was believed to be necessarily controlled by antagonistic sympathetic and parasympathetic ganglia residing at upper lumbar (sympathetic preganglionic innervation) vs lower lumbar and sacral levels (parasympathetic preganglionic innervation) of the spinal cord [47–49]. However, a recent study from Brunet lab challenged this idea by showing that sacral preganglionic neurons innervating pelvic ganglia have a pro-sympathetic profile that does not differ from thoracic sympathetic preganglionic neurons that innervate the sympathetic chain [50]. Specifically, the study showed that the generation of sacral preganglionic neurons depends on the *Olig2* gene, similarly to thoracic preganglionic neurons innervating the sympathetic ganglia and in contrast to the *Phox2b*-dependance of preganglionic neurons innervating parasympathetic ganglia. Additionally, the team showed that ablation of the spinal preganglionic motor neurons did not affect the generation of these pelvic ganglia, proving that they are largely generated from migratory NCCs, similarly to most sympathetic neurons. This embryological argument helped to revisit the original classification of these ganglia formerly known as parasympathetic, as one of the main reasons for the old classification was the use of acetylcholine as their main neurotransmitter. However, as mentioned before, a number of studies have shown that there are cases of sympathetic ganglia using acetylcholine as the main neurotransmitter [40–42]. After the discovery of specifics of pelvic ganglia development, the debates about attributing them to sympathetic, parasympathetic systems or mixed ganglia are still very active [47, 48, 51].

## Chromaffin cells originate from SCPs

Chromaffin cells are neuroendocrine cells that are responsible for the release of catecholaminergic hormones into the blood flow to trigger the “fight-or-fly” response. These cells belong to the sympathetic division of the peripheral nervous system and largely resemble sympathetic neurons. Chromaffin cells differentiate under the control of a transcriptional cascade similar to sympathetic neurons (discussed in more detail below) and use many of the neurotransmitters typical for sympathetic neurons. However, chromaffin cells do not extend neuronal processes, and neither possess classic synaptic machinery. At the same time, they are capable of forming neuro-glandular synapses [52, 53].

There are two major locations of chromaffin cells: the medullary region of the adrenal gland (intra-adrenal) and the transient organ of Zuckerkandl (extra-adrenal). Chromaffin cells of the organ of Zuckerkandl appear in close proximity to the cells of the superior mesenteric ganglion and other local paraganglia. In contrast to this, intra-adrenal chromaffin cells are isolated from the environment by the surrounding adrenal cortex. Embryonic and perinatal chromaffin cells perform an additional function of oxygen-sensing similarly to glomus cells in carotid bodies. This function is believed to be necessary for the first breath initiation and primary adaptation to the extra-uterine life. Chromaffin cells abandon oxygen-sensing function within the first week of postnatal development [54, 55].

**Fig. 2:**
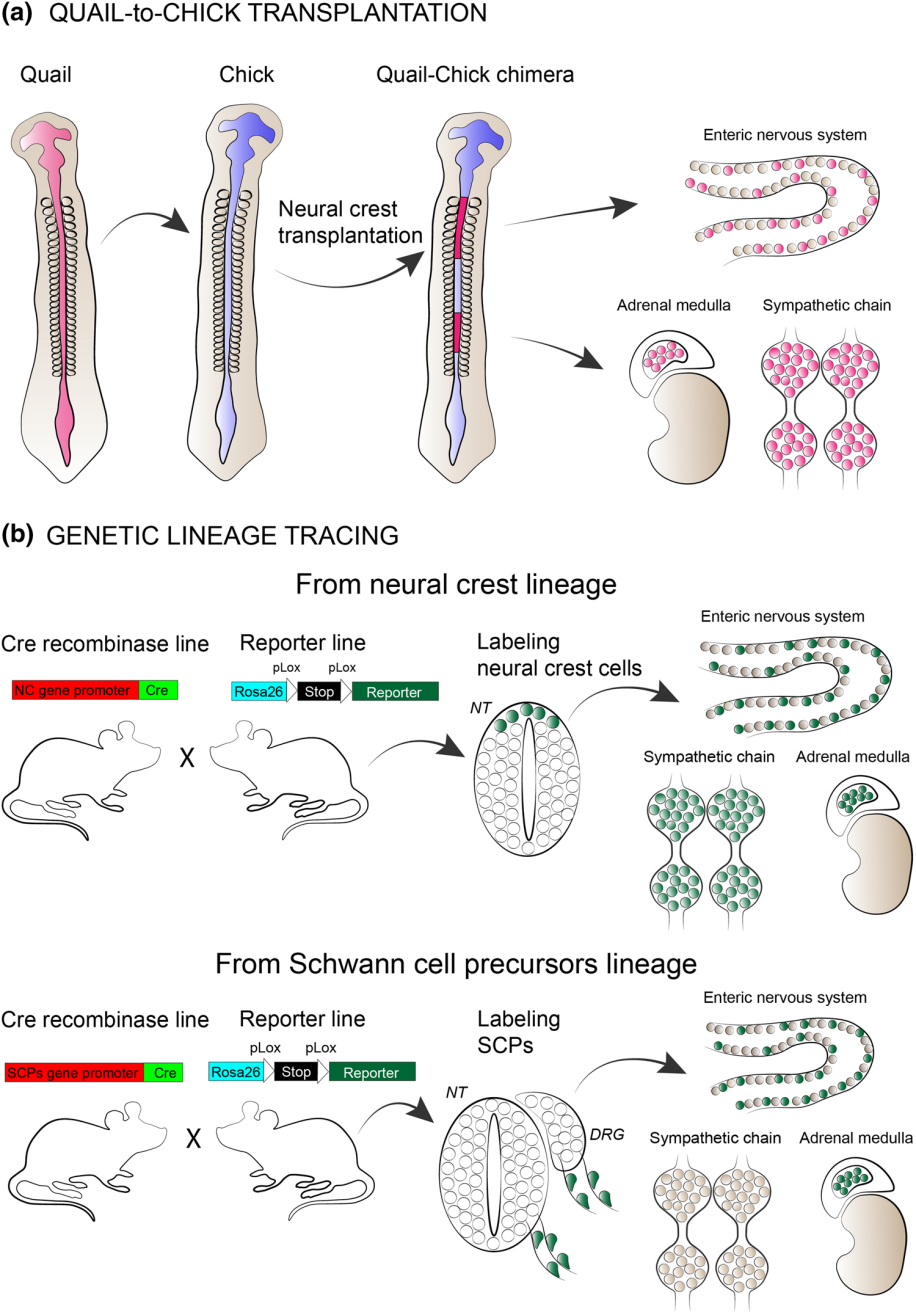
Quail-to-chick transplantation and genetic lineage tracing as tools to discover neural crest cells and Schwann cell precursor to neuronal lineages. **a** quail-to-chick transplantation methodology has revealed the contribution of vagal neural crest to enteric nervous system and limbal neural crest to adrenal medulla and sympathetic chain ganglia. **b** generation of Cre recombinase and reporter transgenic mouse lines enabled lineage specific cell labeling and consecutive discoveries of SCPs contribution to neuronal lineages. *NT* neural tube, *DRG* dorsal root ganglion

The primordium of the adrenal gland starts to coalesce at E11.5 in mouse—long after NCCs complete their migration in this region at E9.5-E10. Previous studies firmly established the neural crest-derived origin of chromaffin cells. Specifically, NCCs between the 18th and 24th somite contributed to formation of adrenal medulla, as shown by heterospecific transplantation of quail neural tube rudiments into developing chick embryo. Subsequently, the quail-specific nucleus structure was identified in chromaffin cells of chick embryos (Fig. 2a) [56, 57]. Morphogens produced by the dorsal aorta are important for proper migration and differentiation of migrating NCCs towards the sympatho-adrenal fate. Among the dorsal aorta-derived morphogens, BMPs do not directly attract the sympatho-adrenal progenitors toward the area of sympathetic ganglia and adrenal gland formation but instead initiate the expression of SDF1 and NRG1 by the para-aortic mesenchyme, which in turn act as chemoattractants for precursor cells. Moreover, BMPs were thought to play a role in the segregation of sympathetic and chromaffin fate, by earlier deactivation of BMP receptors family Smad in sympathoblasts, but prolonged maintenance in chromaffin cells [58]. Upon the migration of the sympatho-adrenal progenitors towards the site of a future primordium, the expression of major transcriptional factors is initiated: *Mash1/Cash1, Phox2A/B, Hand 2, Gata2/3* and *Insm1* (reviewed in [59]).

Previously, adrenal chromaffin cells and sympathetic ganglia were thought to develop from a common population of NCCs based on DiI applications for lineage tracing and numerous in vitro studies directing NCC differentiation into chromaffin and sympathetic neurons upon NGF treatment [60, 61]. The fate segregation was proposed to depend on glucocorticoids and nerve growth factor (NGF) based on the in vitro studies [61]. Later in vivo experiments ensured that glucocorticoids play an important role for the postnatal survival of chromaffin cells and the acquisition of PNMT by some chromaffin cells, but not for chromaffin vs sympathetic fate segregation [62, 63].

The growing block of evidence points to the fact that the precursors of sympathoblasts of the sympathetic ganglia and chromaffin cells are different. For example, chromaffin cells and sympathoblasts differentially express markers of neuronal lineage. Neurofilaments and tyrosine hydroxylase first start to be expressed in sympathoblasts and later at chromaffin cells [64]. The technological advancement in engineering transgenic mice models that allowed cell lineage tracing and lineage specific gene ablation provided important insight into the development of chromaffin cells (Fig. 2b).

**Fig. 3:**
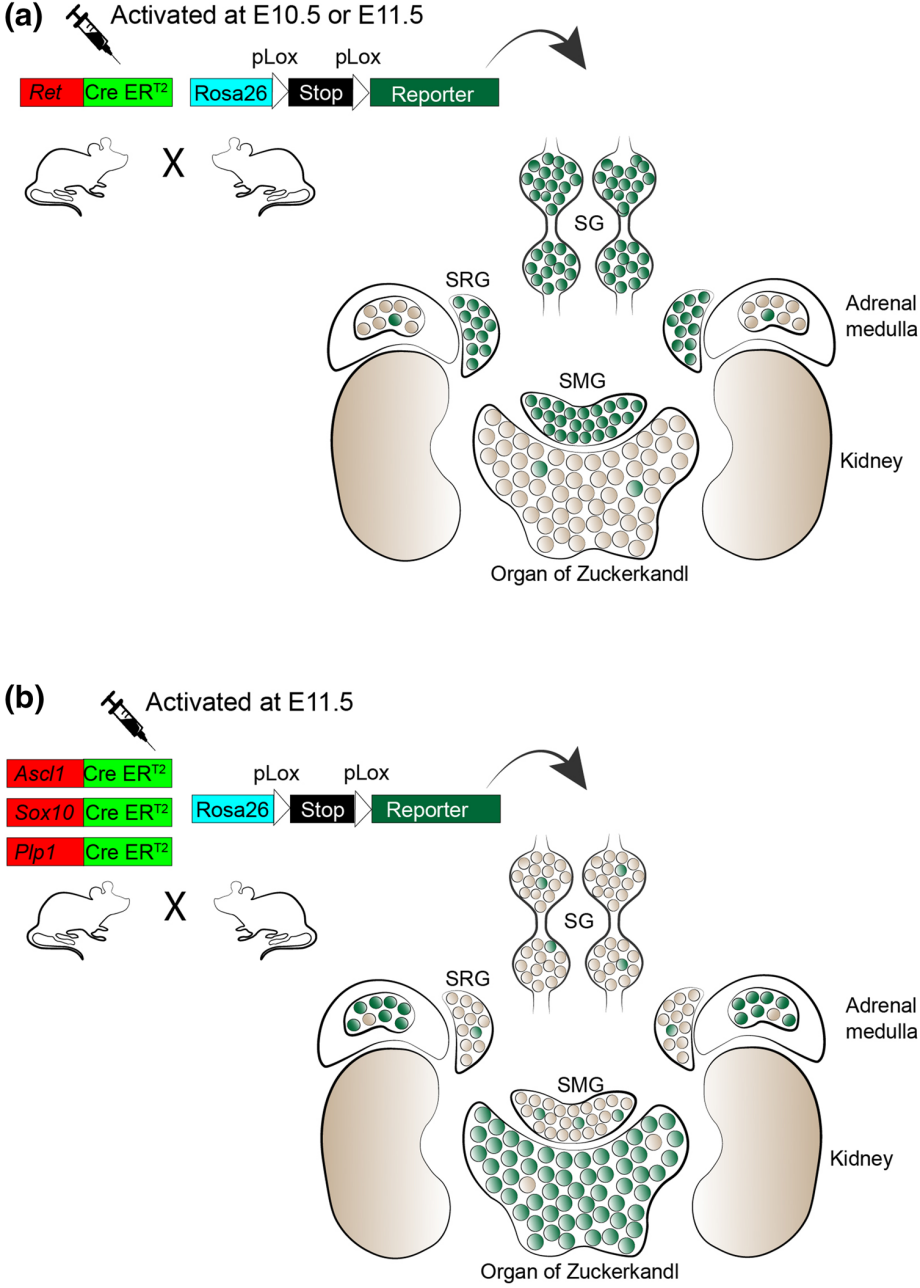
Inducible lineage specific tracing allows to segregate the precursor cells of sympathoblasts and chromaffin cells. **a** induction of Cre recombinase under the control of *Ret* promoter results in labeling of cells in sympathetic ganglia, with minimal contribution to chromaffin cells. **b** induction of Cre recombinase under the control of  *Ascl1* promoter as well as SCPs specific promoters *Sox10* and *Plp1* results in labeling of chromaffin cells, with minimal contribution to sympathoblasts. *SG* sympathetic ganglia, *SRG* suprarenal ganglion, *SMG* superior mesenteric ganglion

According to recent results by Furlan and co-authors, the lineage split between sympathoblasts and chromaffin cells occurs in the vicinity of the adrenal glands before the formation of first chromaffin structures. Lineage tracing with the *Ret*-*CreER*^*T2*^ strain from E9.5 to E11.5 clearly showed that RET^+^ cells give rise to the majority sympathoblasts in paravertebral and suprarenal ganglia, and to only a few chromaffin cells of the adrenal gland or Zuckerkandl organ [18, 19]. In line with these results, lineage tracing using *Ascl1*-*CreER*^*T2*^ demonstrated that ASCL1^+^ progenitor cells generate chromaffin cells, but not sympathoblasts, if recombination is induced at E10.5 (Fig. 3). These results were further supported by SCP- and neural crest-specific inducible Cre lines *Sox10*-*CreER*^*T2*^ and *Plp1*-*CreER*^*T2*^, which revealed a SCP-dependent origin of the majority of chromaffin cells inside of the adrenal medulla and in the organ of Zuckerkandl. In those cases, the genetic recombination resulting in cell labelling occurred after E11.5, with further evidence that labelled cells were SCPs covering preganglionic visceral motor nerves arriving to the location of developing chromaffin structures from the neural tube [18, 19].

**Fig. 4:**
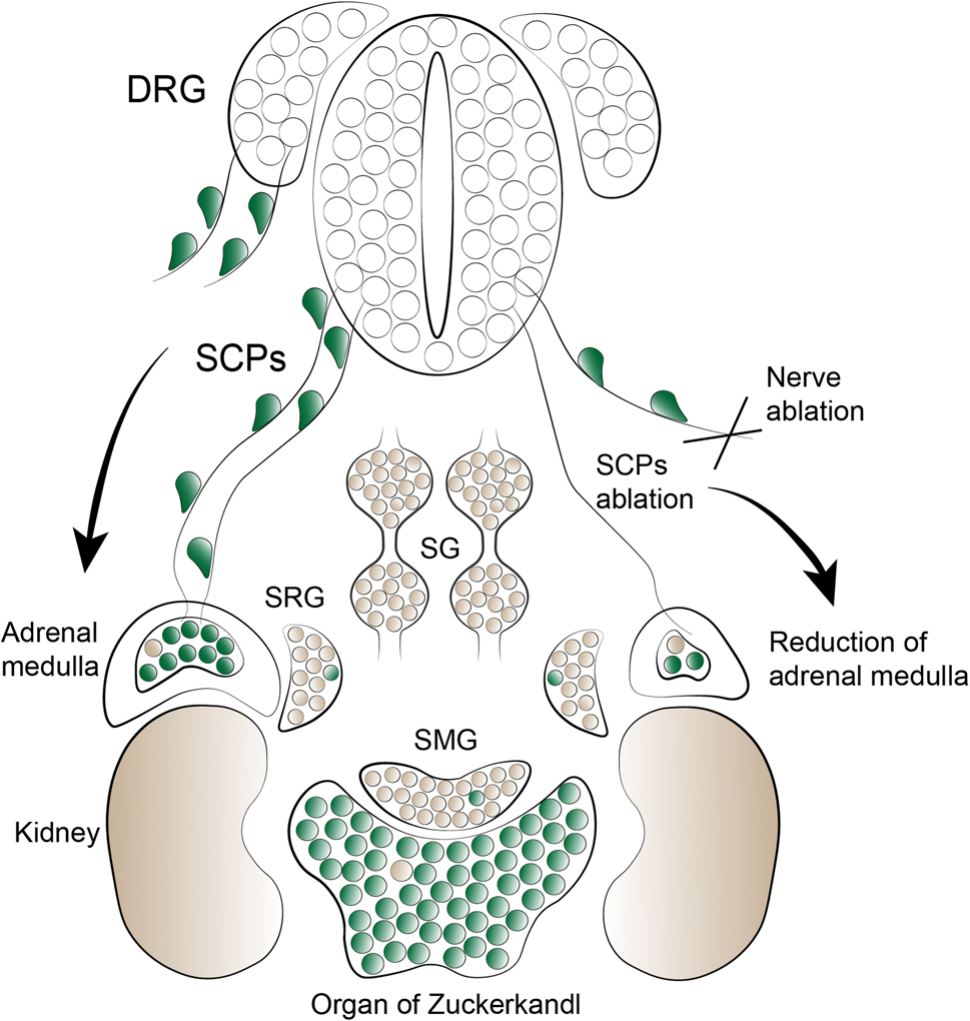
Schwann cell precursors differentiate into chromaffin cells of adrenal medulla and organ of Zuckerkandl. At E11.5 in mice SCPs guided by preganglionic nerves reach the primordium of adrenal gland and differentiate in chromaffin cells. Upon nerve- or SCP-ablation the cells are unable to reach the adrenal gland primordium which results in reduction of the cells building the adrenal medulla. An analogous process governs the development of organ of Zuckerkandl. *DRG* dorsal root ganglion, *SCPs* Schwann cell precursors, *SG* sympathetic ganglia, *SRG* suprarenal ganglion, *SMG* superior mesenteric ganglion

In an attempt to deplete SCPs from the nerves before the onset of chromaffin cell development, Furlan and co-authors crossed *Sox10*-*CreER*^*T2*^ mice with *Rosa26*^*DTA*^ (diphtheria toxin) transgenic strain to induce the death of SOX10^+^ SCPs upon tamoxifen injection. When recombined at E11.5 and E12.5, this experiment resulted in a dramatic depletion of SCPs and chromaffin cells in adrenal medulla. Furthermore, genetic ablation of preganglionic motor nerve fibers (serving as the routes delivering SCPs to the sympatho-adrenal primordium) in *Hb9*–*Cre;Isl2*^*DTA*^ embryos resulted in a substantial decrease of chromaffin cell number in both chromaffin locations (Fig. 4). Consistently, in *Ascl1*^−*/*−^ mice the researchers observed the accumulation of SOX10^−^/PHOX2b^+^/TH^−^ cells along the nerves and the developing primordium and a significant decrease of adrenal medulla and organ of Zuckerkandl chromaffin cells [18, 19].

In an independent chain of experiments, Lumb et al. revealed that genetic ablation of visceral motor neurons in the neural tube results in the dramatic decrease of chromaffin cells in adrenal medulla. Ablation of preganglionic motor neurons by means of *Olig2*-*Cre* mice breeding with *Rosa26*^*DTA*^ strain resulted in loss of 69% of chromaffin cells in the adrenal medulla. Next, Lumb et al. showed that NRP2, a receptor to SEMA3A and SEMA3F, mediates the preganglionic visceral motor axons pathfinding towards the developing adrenal medulla. In *Nrp2*^−*/*−^ and *Sema3f*^−*/*−^ mice, axons that are normally innervating the developing adrenal medulla, abnormally innervated the urogenital ridge. Subsequently, chromaffin cells were found along the misguided axons in an aberrant location. The mis-positioning of chromaffin cells indicates the essential role of axon guidance pathways for achieving the correct anatomic location of tissues containing chromaffin cells [65].

Finally, Furlan et al. performed sc-RNA seq to elucidate the cell fate transition from SCPs to chromaffin cells. When sampled at E12.5 and E13.5, SCPs, chromaffin cells and sympathoblasts form distinct clusters of cells with remarkably different molecular signatures. Some of these clusters, i.e., SCPs and chromaffin cells are connected by a “bridge cell” population with its own gene expression profile including *Htr3a* and other genes. The existence of such “bridge” stage indicates the captured transition of one cell type into another, further proving the SCP-derived origin of chromaffin cells. The scRNA-seq data revealed that the population of SCPs intensely proliferates and fuels the development of adrenergic chromaffin tissue. Chromaffin cells are not proliferatively active at the same stages and slowly resume proliferation only after E14.5 according to the lineage tracing analysis [18].

## Oxygen-sensing cells originate from SCPs

Glomus cells of the carotid body are located at the bifurcation of the carotid artery and are primary oxygen-sensing cells in our bodies. They are responsible for mediating the respiratory response to arterial blood hypoxia and hypercapnia by releasing neurotransmitters dopamine and serotonin to the carotid sinus nerve afferents [66, 67].

Early experiments using chick-quail chimeras revealed that all cells inhabiting the carotid body are neural crest-derived together with part of the carotid artery wall [68]. A more detailed investigation of carotid body growth during hypoxemic conditions revealed that GFAP^+^ glial cells resident in the organ are the source of increased proliferation and growth [69]. Later lineage tracing experiments revealed that the carotid body has a composite origin and it is partly derived from local SCPs. When the recombination was induced in the embryos carrying SCP- and neural crest-specific *Plp1*-*CreER*^*T2*^ at E12.5, PLP1^+^ progenitors gave rise up to 60% of all glomus cells in the carotid body [20]. Another portion of the carotid body originated from the neural crest-derived neuroblasts, which transiently populate the nodose ganglion. This was validated by grafting the vagal neural crest region from the GFP^+^ transgenic donor chick embryo into the non-transgenic host embryo to monitor the cell fate later in development. After grafting, the neural crest-derived cells were first observed in the nodose ganglion closer to the carotid body. Soon after, these cells emigrated towards the carotid body forming the peripheral part of the organ. The migrated GFP^+^ cells started to express TH and showed the presence of serotonin unlike nodose ganglion neurons [20].

## A significant part of the enteric nervous system is SCP-derived during embryonic and postnatal stages

The enteric nervous system (ENS) is the largest compartment of the peripheral nervous system and is composed of two neuronal networks: the myenteric and submucosal plexus, spanning from the esophagus to stomach and intestine. The ENS is responsible for the control of intestinal motility and secretory functions (reviewed in [70]) as well as involved into complex relationships with microbiota [71, 72]. The cell type composition of ENS is still under investigation despite significant recent progress in this direction [73–75].

Even though the colonisation of the prospective foregut starts as early as E9.5 in mouse by vagal NCCs, the ENS keeps developing during embryonic and early postnatal stages to accommodate for the growth of digestive tract. Logically, such a long span of a developmental period and following growth requires a long-lasting source of precursor cells.

**Fig. 5:**
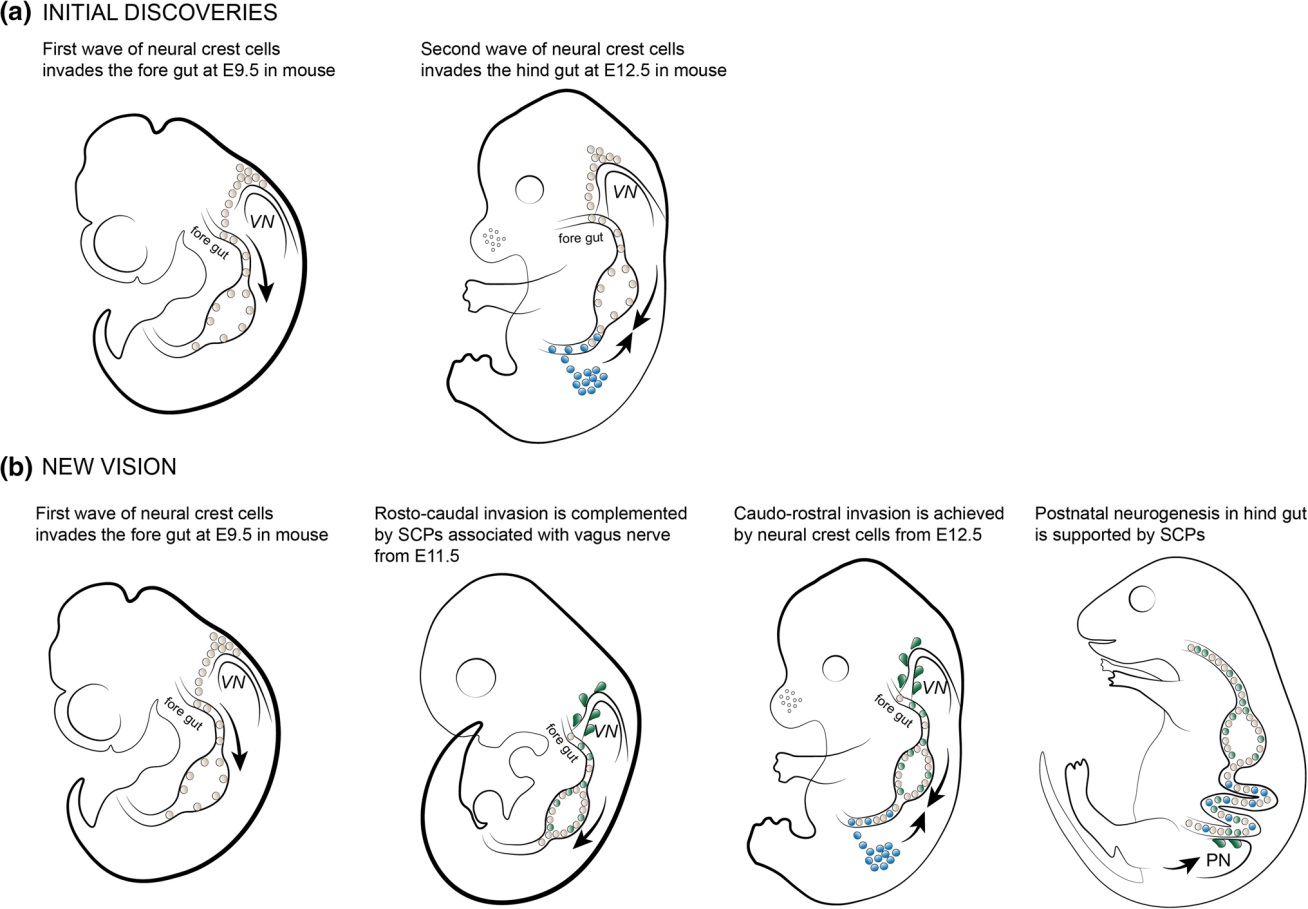
Recent update in the understanding of enteric nervous system development. **a** initial experiments revealed that vagal neural crest contributes to the entire enteric nervous system and sacral neural crest contributes to the enteric nervous system in the most caudal part of the gut. **b** late genetic manipulations in mice revealed the role of SCPs in building the enteric nervous system during embryonic and postnatal development. *VN vagus* nerve, *PN* pelvic nerve, *SCPs* Schwann cell precursors

The first evidence of NCCs giving rise to the cells of the ENS came from a study in which surgical removal of post-otic neural crest in chick embryos was performed, leading to reduced development of ganglia in lungs, heart, esophagus and gastrointestinal tract [76]. Later experiments on chick-quail chimeras showed that vagal NCCs (somites 1-7) are precursor cells for the entire avian ENS spanning from the crop to large intestine (Fig. 5a). At the same time, sacral neural crest at the level of 28th somite gave rise to post-umbilical gut ganglion [77]. The observation of developmental dynamics disclosed that vagal NCCs first accumulate in the caudal branchial arches at E3.5 in chick and enter the foregut (including esophagus, stomach, duodenum) to migrate rostro-caudally toward the cecal–colorectal junction by E7 in chick. During later stages, sacral NCCs start migrating caudo-rostrally at E4 in chick and colonize the gut up to the level of the bile ducts in duodenum. Therefore, the avian ENS of the hindgut consists of the progenies of vagal and sacral NCCs with the increasing contribution of sacral NCCs in the rostro-caudal direction reaching 17% in myenteric plexus and 1.3% in submucosal plexus in the most caudal part of the gut [78]. The detailed study of dynamic of enteric NC migration revealed that vagal NC-derived cells migrate as a population and cell-to-cell contacts are important for the propagation of the leading group of cells along the enteric mesenchyme in rostro-caudal direction [79]. Sacral NCCs have a limited potential to migrate, perhaps because of lower expression of Ret in comparison to vagal NCCs [80]. The sacral NC first forms the pelvic ganglia and only later start invading the hind portion of the gut, after the vagal NCCs have already populated it (Fig. 5a) [81] (reviewed in [82]). During the development of intrinsic gut innervation from E10.5 to E11.5, the extrinsic innervation of the intestinal wall develops, carrying high numbers of SCPs along the extrinsic nerves [83, 84].

Among the major factors playing a key role in ENS development are RET, GDNF and GFRα1. The receptor tyrosine kinase RET couples to GFRα1 in a GDNF-mediated manner to initiate downstream signaling pathways. RET/GFRα1/GDNF signaling plays an important role in the development and survival of peripheral autonomic, sensory neurons as well as dopamine neurons in central nervous system. Downstream of this pathway ERK, PI3P/AKT, MAPK and JNK signaling pathways play various roles in cell survival and proliferation (reviewed in [85]). Moreover JNK, MEK and cAMP dependent cascades play critical roles in speed and directionality of the migration of ENS progenitors [86]. Mouse models lacking *Ret* [87–89], *Gdnf* [90–92] or *Gfrα1* [93] demonstrate aganglionic small and large intestine; however, none of them shows a complete lack of enteric neurons. Most importantly, vagal NCCs in these models cease their migration and undergo cell death before colonizing caudal parts of the intestine. Next, in *Ret*^−*/*−^ and *Gfra1*^−*/*−^ perinatal embryos, a few enteric neurons were found in the submucosal layer of the intestine, distal from stomach and being associated with extrinsic innervation. Such neurons were completely absent from the areas of intestine that do not have any extrinsic innervation [15]. These observations suggested that there is an additional source of enteric neurons, independent of RET/GFRα1/GDNF signaling and dependent on extrinsic innervation. Additionally, tamoxifen-induced tracing initiated at E12.5 in *Sox10*-*CreER*^*T2*^; *Rosa26*^*YFP*^ mice resulted in tracing of 30-40% of enteric neurons when inspected at postnatal stages [17]. Taking in consideration that there is no migratory crest after E10.5, SCPs were hypothesized to be a probable additional source of enteric neurons, pre- and postnataly, in addition to the early waves derived from the vagal and sacral neural crest [17].

To analyse if enteric neurons in *Ret*^−*/*−^ and *Gfrα1*^−*/*−^ mice were indeed derived from SCPs, Uesaka and colleagues utilised the following strategy: *Dhh*-*Cre* mice (*Dhh* is expressed specifically in SCPs) were crossed with *Gfrα1*^−/−^ mice caring the *Ret*^*fl*−*CFP/*+^ reporter allele (upon the recombination, one allele of *Ret* is inactivated, and CFP is activated to label the cells [94]). Elimination of *Gfra1* did not limit SCPs from generating submucosal neurons, thus, strongly suggesting their role in generating enteric neurons in the absence of enteric NCC (ENCC) [15].

The analysis of distribution of traced cells in *Dhh*-*Cre*;*Ret* ^*fl*−*CFP/*+^ 1-month-old mice showed that SCP contribution to the neurons of small and large intestine differed substantially. Only 5% of neurons in small intestine submucosal ganglia occurred to be SCP-derived. Similarly, SCP-derived neurons were barely found in the myenteric plexus of the small intestine [15].

In the large intestine, around 20% of neurons are SCP-derived, populating both myenteric and submucosal layers. Most SCP-derived neurons express key enteric neurotransmitters *Calb2* and n*Nos* and form synapses with immediate neural crest-derived neurons, indicating the efficient integration in the intrinsic neural circuits [15]. Furthermore, lineage tracing in *Sox10*–*CreER*^*T2*^; *Rosa26*^*Confetti*^ reporter mice induced at E12.5 and analysed in adult stages revealed SCP-derived clones being composed of only neuronal or only glial cells, or neuro-glial mixtures. The identified clones occupied a compact space indicating a limited potential for migration of the progenitor cells within myenteric and submucosal layers. The clonal organisation of the ENS has implications in sister cell-neurons responding to external stimuli in a synchronized manner in comparison with non-related cells [95].

Additionally, the inspection of rostral levels of the digestive system revealed a significant proportion of the esophageal neurons being SCP-derived [16]. SCPs reach the developing esophagus via the incoming *vagus* nerve, which is found covered by SOX10^+^/PHOX2B^+^/PLP1^+^/CDH19^+^ cells at E11.5. This suggestion has been validated by a set of genetic ablation and lineage tracing experiments. For instance, the partial ablation of the *vagus* nerve (achieved by the expression of a toxic version of ASIC2A under the control of *Phox2a* promoter) led to a 36% reduction of SOX10^+^ cells and PHOX2B^+^ neuronal precursors in the esophagus. Experimental disruption of the ERBB3-NRG1 signalling inhibited SCP migration along the *vagus* nerve, which resulted in esophageal ganglia being depleted of neurons by half [16]. Therefore, during embryogenesis, the esophageal ENS is derived from vagal NCCs starting to invade the wall of esophagus at E9.5 and being complemented by SCPs from the *vagus* nerve from E10.5 and onwards (Fig. 5b).

Altogether, these findings show that initial waves of enteric neurogenesis originate from the vagal and sacral neural crest and are being doped at later stages by a wave of SCP-derived neurogenesis. The enteric neurons, which are generated at different stages and from different immediate origins, integrate into fully functional units. Interestingly, SCP-driven enteric neurogenesis is evident in a postnatal ENS injury model, where SOX10^+^ cells give rise to the enteric neurons in adult mice during regeneration [17]. The most recent finding indicates that in post-embryonic zebra fish SCPs associated with the extrinsic gut innervation but not the enteric resident precursor cells are responcible for de novo neurogenesis [96].   

## Clinical implications of SCP multipotency

SCPs are multipotent nerve-associated cells, giving rise to neuronal or neuroendocrine subpopulations of the peripheral nervous system. Parasympathetic, sympathoadrenal and enteric domains demonstrate various degree of SCP-derived neurons. Abnormalities of SCPs migration and differentiation towards these neuronal cell types might result in specific congenital diseases associated with SCP-derived neurogenesis.

The sympathoadrenal system including chromaffin and sympathetic cells as well as oxygen-sensing cells of carotid body is a well-known source of several malignancies. For instance, sympatho-adrenal cells might be the source of an early childhood cancer neuroblastoma [97] and an adult cancer pheochromocytoma [98]. The extra-medullary sympatho-adrenal cells of the organ of Zuckerkandl disintegrate in healthy individuals by the age of 3 years. However, failure in this process might cause tumors during adulthood [99, 100]. Additionally, oxygen-sensing glomus cells give rise to paraganglioma in adults [101, 102].

Neuroblastoma is the solid extra-cranial tumor associated with genetic defects in transcriptional factors MYCN, ALK [103], and PHOX2B [104, 105], which play important role in sympatho-adrenal lineage development (reviewed in [106]). There are several ways to classify neuroblastoma tumors. In 2009, International Neuroblastoma Risk Group (INRG) Task Force has proposed a system, which included age, histologic category, grade of tumor differentiation, the status of the MYCN oncogene, chromosome 11q status, and DNA ploidy as characteristics of the tumors. This system is currently used in clinical practice to diagnose and plan the future therapy [107]. A complementary study of gene expression profiles identified by microarrays further characterized the types of tumors and classified additional tumor types associated with poor prognosis [108]. Based on this recent classification, low risk tumors belong to the type 1 and are characterized by triploid DNA content, numeric alterations, and high expression of TRKA [109, 110]. Intermediate risk tumors do not have MYCN alterations, but demonstrate typical for neuroblastoma chromosomal aberrations: deletion of 11q and gain of 17q, which help to classify the tumors as type 2A. Type 2B is a high-risk neuroblastoma and presents many similarities to type 2A with a key difference of carrying *Mycn* gene amplification.

One of the most enigmatic features of neuroblastoma is the phenomenon of spontaneous regression (referred to as type 4S), which can happen in about 10% of detected cases [111]. Moreover, there is a specific type of neuroblastoma with metastases in skin, liver and bone marrow, which also falls in the group of self-regressing tumors. Such spontaneous regression sometimes leads to ganglioneuroma—a benign type of tumor (reviewed in [112].

Several studies pointed to the fact that multipotency is retained in some neuroblastoma tumors, which hypothetically might be explained by the SCP-related developmental origin of chromaffin cells and some sympathoblasts. Neuroblastoma tumors are mixed tumors, composed of an adrenergic component identified by the expression of *Phox2b, Hand2*, *Gata3* and a mesenchymal component (also referred to as a NCC-like component) identified by the expression of *Prrx1, Vim, Snai2, Fosl1/2, Jun* and others [113, 114]. Although the genetic abnormalities observed in the cells composing neuroblastoma tumors are largely uniform, the super-enhancer-associated transcription factor networks and corresponding transcriptional profiles are different for adrenergic-like and mesenchymal populations. Furthermore, there is evidence of adrenergic-to-mesenchymal and mesenchymal-to-adrenergic transitions, which may result in unwanted relapses and therapy tolerance observed in neuroblastoma [113–115]. Finally, even though neuroblastoma is hypothesized to originate from the migratory and sympatho-adrenal neural crest, there might be clinical cases, where abnormal Schwann cell lineage causes specific neuroblastoma and pheochromocytoma subtypes.

Another relevant tumorigenic disease is neurofibromatosis type 1 (reviewed in [116]). The mutation in *Nf1* gene causes cells belonging to the Schwann cell lineage to be susceptible to benign tumor formation [117, 118]; however, to form neurofibromas, the *NF1* deficient microenvironment is also necessary [119]. In *Dhh*-*Cre;Nf*^*fl/fl*^ and *Mpz*-*Cre;Nf1* ^*fl/fl*^ mice with *Nf1* gene was removed from the Schwann cell lineage, the development of plexiform neurofibromas, dermal neurofibromas, and pigmentation was observed [120, 121]. Therefore, one of the possible cells of origin of neurofibromatosis type 1 is SCP in their transition to immature SCs. Within neurofibromas, different cell types including NF1-deficient Schwann cell-like cells, fibroblasts, perineurial-like cells, neural cell elements (mainly processes) [122] and recruited mast cells [123] co-exist and grow. There is a need to better understand what triggers differentiation of SCPs into these abnormal cell types to prevent the further development of neurofibromatosis.

Finally, the failure of precursor cells to reach and colonize the gut is the reason for Hirschsprung’s disease characterized by the lack of innervation in the distal portion of the bowel. Consistently, in case of Hirschsprung’s disease, the external nerves innervating the gut are abnormally thick with excessive NGF levels and accumulation of p75^+^/S100^+^ glial cells [124, 125] resembling SCPs. Currently, the surgical removal of aganglionic gut is a widely used treatment for the disorder. However, there are challenges in the surgery if a substantial portion of gut is de-innervated. Therefore, the need for an alternative therapy persists. In principle, SCPs and SCs can be harvested from the healthy portion of the gut, expanded in a neurosphere culture and subsequently transplanted into the aganglionic portion of the gut for regenerating local ENS [125, 126]. Moreover, the presence of SCPs associated with extrinsic innervation in the gut of Hirschsprung’s disease patients raises the possibility [127] of a therapy based on the direct induction of neuronal differentiation of these precursor cells within the gut walls [125].

## Concluding remarks

The last decade of research significantly expanded our insights into the multipotency and key roles that SCPs play in the body. Starting from a non-mainstream finding of SCP-derived melanocytes in 2009 [10], the field underwent inflation and generated the knowledge of SCPs contributing to numerous cell types previously known as descendants of NCCs. Today, SCPs are rather seen as a continuation of NCCs that occupies the nerves and utilizes the innervation as a navigating route. Because spatial and temporal heterogeneity of SCPs still remains elusive, further innovations in single-cell epigenetic, genomic and spatial transcriptomic technologies will largely advance our understanding of regional differences in SCPs including their local differentiation potential.

SCPs might be a perfect adaptation to accommodate for a fast embryonic growth and arising deficits of free migration (the concept is proposed in [25]). In line with this, it is tempting to suggest that a small pool of undifferentiated SCPs remains associated with nerves and can serve as a stem cell population recruited upon the need of tissue regeneration or as in the case of the carotid body additional growth as an adjustment to chronic hypoxia. SCs are known to de-differentiate in case of nerve injury, re-enter the cell cycle and assist in the axon regeneration (for review [128]). Therefore, SCs from patient-derived skin biopsies could be reprogrammed and used in regenerative strategies via activation of SCP-like multipotency.
